# Biodegradable polymeric stents for vascular application in a porcine carotid artery model: English version

**DOI:** 10.1007/s00772-015-0011-z

**Published:** 2015-03-18

**Authors:** S. Kischkel, N. Grabow, A. Püschel, B. Erdle, M. Kabelitz, D.P. Martin, S.F. Williams, I. Bombor, K. Sternberg, K.-P. Schmitz, W. Schareck, C.M. Bünger

**Affiliations:** 1 Klinik und Poliklinik für Allgemeine, Thorax-, Gefäß- und Transplantationschirurgie, Universitätsmedizin Rostock, Schillingallee 70, 18057 Rostock, Germany; 2 Institut für Biomedizinische Technik, Universitätsmedizin Rostock, Rostock, Germany; 3 TEPHA, Inc., Lexington, USA; 4 Institut für Diagnostische und Interventionelle Radiologie, Universitätsmedizin Rostock, Rostock, Germany; 5 Klinik für Gefäßmedizin, Vivantes Klinikum Humboldt, Berlin, Germany

**Keywords:** Biodegradable stents, Poly(L-lactide), Poly(4-hydroxybutyrate), Common carotid artery, Atorvastatin, Biodegradierbarer Stent, Poly(L-Lactid), Poly(4-Hydroxybuttersäure), A. carotis communis, Atorvastatin

## Abstract

Over the past years the development of biodegradable polymeric stents has made great progress; nevertheless, essential problems must still be solved. Modifications in design and chemical composition should optimize the quality of biodegradable stents and remove the weaknesses. New biodegradable poly-L-lactide/poly-4-hydroxybutyrate (PLLA/P4HB) stents and permanent 316L stents were implantedendovascularly into both common carotid arteries of 10 domestic pigs. At 4 weeks following implantation, computed tomography (CT) angiography was carried out to identify the distal degree of stenosis. The PLLA/P4HB group showed a considerably lower distal degree of stenosis by additional oral application of atorvastatin (mean 39.81 ± 8.57 %) compared to the untreated PLLA/P4HB group without atorvastatin (mean 52.05 ± 5.80 %). The 316L stents showed no differences in the degree of distal stenosis between the group treated with atorvastatin (mean 44.21 ± 2.34 %) and the untreated group (mean 35.65 ± 3.72 %). Biodegradable PLLA/P4HB stents generally represent a promising approach to resolving the existing problems in the use of permanent stents. Restitutio ad integrum is only achievable if a stent is completely degraded.

## Abstract (de)

Obwohl die Entwicklung biodegradierbarer Polymerstents in den letzten Jahren große Fortschritte gemacht hat, verbleiben entscheidende noch zu lösende Probleme bestehen. Veränderungen im Design und in der chemischen Zusammensetzung sollen die Eigenschaften biodegradierbarer Stents optimieren und ihre Schwächen beseitigen.

Der neue PLLA/P4HB-Stent konnte erfolgreich endovaskulär in die A. carotis communis von 10 Landschweinen implantiert werden, bei vergleichbaren mechanischen Eigenschaften zu 316L-Metallstents als Kontrollgruppe. 4 Wochen nach Implantation erfolgte eine Angio-CT zur Stenosegradbestimmung. Es zeigten sich deutlich höhere Stenosegrade in der PLLA/P4HB-Stent-Gruppe (52,05 ± 5,80 %) gegenüber der Metall-Stent-Gruppe (35,65 ± 3,72 %). Durch die orale Gabe von Atorvastatin konnte der distale Stenosegrad nach NASCET in der PLLA/P4HB-Stent-Gruppe (39,81 ± 8,57 %) auf das Niveau der Metall-Stent-Gruppe (44,21 % ± 2,34) gesenkt werden.

Der PLLA/P4HB-Stent stellt einen vielversprechenden Ansatz zur Lösung der bestehenden Probleme beim Einsatz permanenter Stents dar. Nur wenn sich ein Stent vollständig auflöst, ist eine Heilung im Sinne einer Restitutio ad integrum möglich.

## Introduction

The ability to treat vascular disease using interventional approaches, as well as and the development of endovascular treatment techniques have made the use of stents routine. Compared with single-vessel dilatation, as in percutaneous transluminal angioplasty (PTA), they prevent in particular acute vascular recoil by providing scaffolding, thereby effectively reducing the restenosis rate [1]. Alongside permanent bare metal stents (BMS) of various designs, alloys and applications, biodegradable polymer stents are playing an ever more important role. Compared with conventional permanent BMS, they have the advantage of preventing chronic foreign-body reactions and enabling secondary vascular surgery in previously stented areas.

The aim of this article is to present the results from an animal model following endovascular implantation of an advanced and improved biodegradable PLLA/P4HB stent compared with a permanent 316L metal stent for use in the peripheral vasculature. The effect of an orally administered statin is also investigated.

## Materials and methods

### PLLA/P4HB stents

The biodegradable polymer stents used in this study were developed, produced and tested in vitro at the Institute for Biomedical Technology at the University Medicine Rostock [2].

**Fig. 1:**
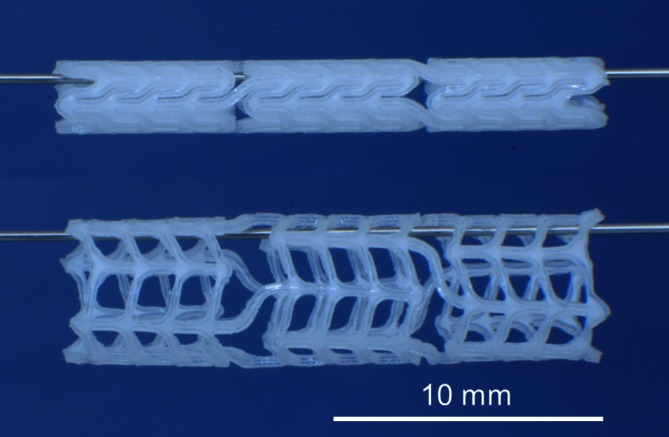
The biodegradable PLLA/P4HB stent unexpanded (*top*) and expanded (*bottom*)

In brief, the stents were produced in an automated, PC-assisted immersion process and cut from tiny tubes (D_i_ = 2.2 mm, D_a_ = 2.7 mm) using a CO_2_ laser [2]. The stents were made from a blend of poly(l-lactide) and poly(4-hydroxybutyrate) (PLLA/P4HB). The chemical composition of their basic substance consisted of 78 % PLLA (Resomer L214, Boehringer, Ingelheim, Germany) and 22 % P4HB (TephaFLEX, Tepha, Inc., Lexington, MA, US). Fig. 1 shows the PLLA/P4HB stent with its nominal dimension of 5.0 × 20 mm (wall thickness: 250 μm).

### Metal stents

Non-biodegradable stents made from medical-grade stainless steel (316L) and coated with amorphous silicon carbide (Dynamic, Biotronik SE & Co. KG, Berlin, Germany) with a nominal dimension of 5.0 × 25 mm (wall thickness: 160 μm) were used as controls.

### Animal preparation

The experiments were approved by the Mecklenburg-Vorpommern Regional Office for Agriculture, Food Safety and Fisheries in accordance with the regulations on animal experiments set out in §8 par. 1 of the German Animal Welfare Act. The study used 10 healthy female domestic swine weighing 26.1 ± 1.7 kg.

Food was withheld for at least 12 h preoperatively, beginning on the day prior to surgery; drinking water remained available at all times. The experimental animals were premedicated with azaperone [Stresnil®, 5 mg/kg body weight (BW); Janssen-Cilag, Neuss, Germany), ketamine (40 mg/kg BW; Bela-Pharm, Vechta, Germany), midazolam (Dormicum®, 0.2 mg/kg BW; Hoffmann La Roche, Grenzach/Wyhlen, Germany) and atropine sulfate (0.01 mg/kg BW; Germany)] via intramuscular injection in the neck area. The animals were intubated and received volume-controlled mechanical ventilation (Ventilog 2, Dräger, Lübeck, Germany) with a 40 % oxygen–1.2 Vol% isoflurane (Abbott GmbH & Co KG, Wiesbaden, Germany) mixture. The animals were anesthetized during surgery with fentanyl (0.25 mg/h i.v., Fentanyl®-Janssen, Janssen-Cilag, Neuss, Germany) and pancuronium (10 mg/h i.v., Pancuronium Hikma®, Hikma Pharma, Klein-Winternheim, Germany) administered to the ear via a 20-G indwelling catheter (Vasofix®, Braun-Melsungen AG, Germany). Fluid substitution was performed with Ringer’s lactate (Sterofundin®, Braun-Melsungen AG, Germany). Ampicillin/sulbactam (1.5 g; Unacid®, Pfizer, Karlsruhe, Germany) was administered preoperatively as a single-shot antibiotic prophylaxis.

One group of five animals also received 60 mg/day atorvastatin (Sortis, Pfizer, Germany) orally in addition to food, beginning 5 days prior to surgery and lasting until the study ended.

### Stent implantation

Following surgical incision in the lower left quadrant of the abdomen, the left common iliac artery was exposed. A 6-F sheath (Radiofocus® Introducer, Terumo, Germany) was introduced by puncture. After probing the target vessel, an 8-F introducer system (Flexor® Tuohy-Borst Sidearm Introducer, Cook, Germany) was inserted over a guidewire (Rosen Wire, Cook, Germany). The stents were placed in the carotid artery under X-ray fluoroscopy and then balloon-expanded.

**Fig. 2:**
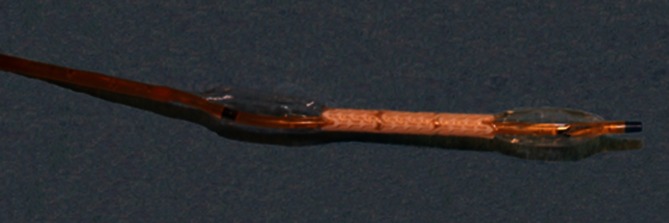
A PLLA/P4HB stent mounted on a PTA balloon catheter and predilated extracorporeally with 1 bar

As a commercial stent-catheter system (Dynamic 5/25/130, Biotronik SE & Co. KG, Berlin, Germany), the metal stents were rapidly inflated with a pressure of 9 bar. The biodegradable PLLA/P4HB stents were implanted using a 5.0 × 40 mm PTA balloon catheter (Passeo-35: 5/40/130, Biotronik SE & Co. KG, Berlin, Germany). To this end, the PLLA/P4HB stents were mounted manually on the balloon catheter, predilated extracorporeally with 1 bar (Fig. 2) and dilated in situ at the target site with a pressure of 8 bar for 1 min. The side of the common carotid artery (CCA) on which a PLLA/P4HB stent or a 316L stent was implanted was left to chance. Following stent implantation, the balloon catheters were deflated and withdrawn. Once implantation was complete, layer-by-layer wound closure was performed.

### Antithrombotic medication

The animals received continuous intravenous heparin (50 IU/kg per hour) during the entire intervention. Oral dual antiplatelet drugs comprising 250 mg acetylsalicylic acid (ASA; Aspirin®, Bayer, Leverkusen, Germany) and 75 mg clopidogrel bisulfate (Plavix®; Sanofi-Synthelabo, Berlin, Germany) were administered daily from postoperative day 3 until the end of the experiment.

### Investigation methods

Basic parameters (BW, heart rate, arterial RR, pO_2_, pCO_2_, hemoglobin, hematocrit, partial thromboplastin time, C-reactive protein) were routinely monitored in all animals pre-, intra- and postoperatively.

Invasive intra-arterial angiography (digital subtraction angiography, DSA) was performed using a high-pressure injector (Medrad, Avida Injector, Germany) to assess vessel size before, and vessel reaction after, stent dilatation.

Contrast-enhanced computed tomography (CT) examinations were performed on the previously premedicated animals at the Radiological Unit of the University Medicine Rostock 4 weeks following stent implantation to assess stent patency and for the purposes of flow measurement.

### Statistical analysis

Expansion length as well as stent and vessel diameter were measured from the CT data using the Leonardo Workstation (Siemens Medical Solution, Erlangen, Germany). The distal degree of stenosis was calculated according to the North American Symptomatic Carotid Endarterectomy Trial (NASCET), whereby the vessel inner diameter in the region of plaque-induced vessel stenosis was substituted by the stent inner diameter (ID_S_):

(1) $$ {\rm{NASCET}}{\mkern 1mu} [\% ] = ({\rm{I}}{{\rm{D}}_{\rm{G}}} -{\rm{I}}{{\rm{D}}_{\rm{S}}})/{\rm{I}}{{\rm{D}}_{\rm{G}}}\cdot100 $$

Using statistical methods (SigmaPlot Version 12.0), the data were given as a mean value ± standard deviation(SD). Differences between two groups were compared using Student’s t-test for unpaired samples. The Friedmann test was used for more than two related samples, whilst the Kruskal-Wallis one-way analysis of variance was used for more than two unrelated samples. A p-value of less than 0.05 was considered statistically significant.

## Results

All stents could be successfully implanted in the CCA in experimental animals. All animals survived until the end of the study period. No wound healing disorders were observed during this time. The animals exhibited a weight gain of 12.1 ± 2.3 kg within the 4-week period.

**Fig. 3:**
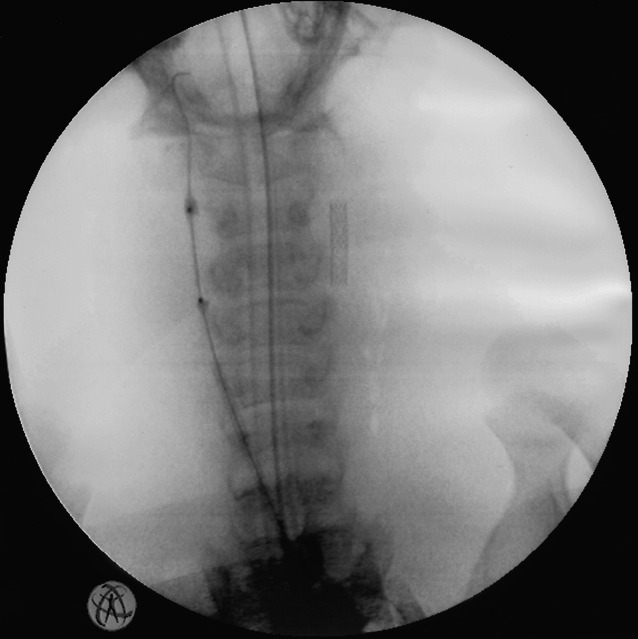
X-ray showing early dilatation of a PLLA/P4HB stent (*left*) compared with the implanted 316L metal stent (*right*) in situ (porcine carotid artery)

Following successful implantation, all stents showed good dilatation and patency on final angiography. None of the stents had collapsed or showed signs of relevant recoil. All stents had remained at the target site and patent following deflation and withdrawal of the balloon catheter. Figure 3 shows an X-ray demonstrating early dilatation in a polymer stent compared with a metal stent.

**Fig. 4:**
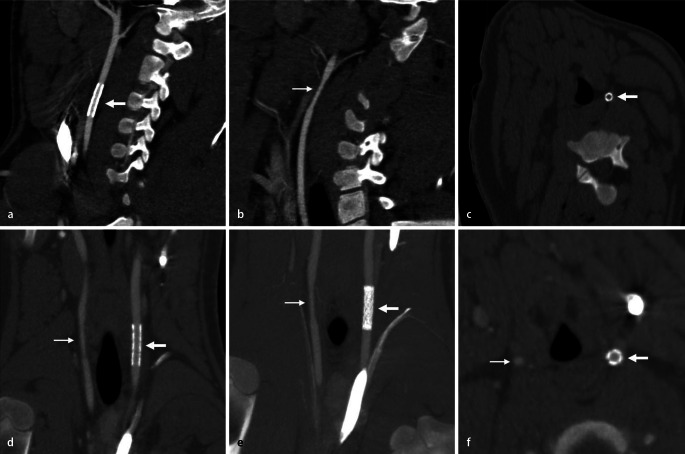
Representative CT images of the common carotid artery, fitted with both types of stent, in the sagittal (**a**, **b**), coronal (**d**, **e**) and axial (**c**, **f**) planes. **c** Without contrast medium. **e** Multiplanar reconstruction. *Thin arrows* point to a PLLA/P4HB stent, *thick arrows* to a 316L stent

At 4 weeks following implantation, all carotid stents were open and permeable, irrespective of the side of the CCA on which stents had been placed. Figure 4 shows representative CT images of the CCA fitted with both types of stent in sagittal, coronal and axial planes.

**Fig. 5:**
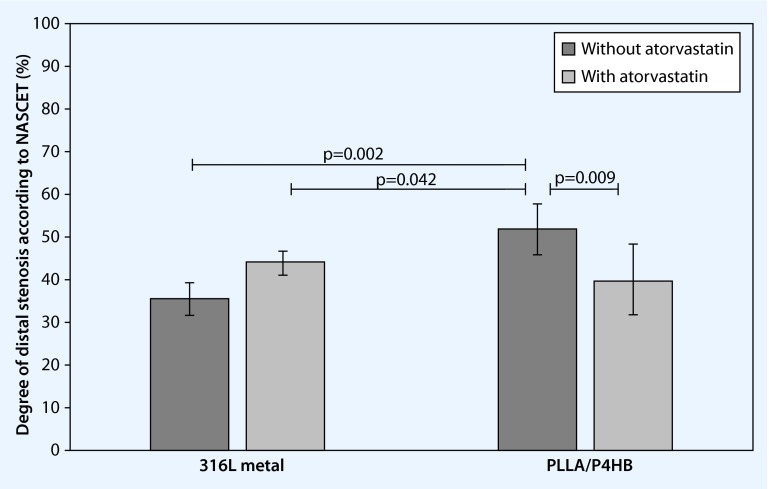
Degree of distal stenosis according to NASCET (%) 4 weeks following endovascular stent placement in the common carotid artery. Data are presented as mean value ± SD. The Kruskal-Wallis test, followed by the Student-Newman-Keuls test, was used to compare the groups

A significantly higher degree of stenosis according to NASCET was seen in the CCA in the group with PLLA/P4HB stents compared with the metal stent group (52.05 ± 5.80 % vs. 35.65 ± 3.72 %; *p* < 0.002) 4 weeks following endovascular implantation. The distal degree of stenosis in the PLLA/P4HB stent group could be reduced to the level of the metal stent group by means of oral application of atorvastatin (39.81 ± 8.57 % vs. 44.21 % ± 2.34; *p* > 0.234). No differences were seen between animals receiving and those not receiving atorvastatin in the metal stent group (*p* > 0.07). In contrast, in the PLLA/P4HB stent group, a significantly lower degree of distal stenosis was seen in animals receiving atorvastatin compared with those not (*p* < 0.009) (Fig. 5).

The vessel lumen distal and proximal to the stent showed no differences between the two stent groups, irrespective of whether or not the animals had received atorvastatin (Tab. 1). No differences between nominal length (316L: 25 mm; PLLA/P4HB: 20 mm) and expanded length (316L: 25.27 ± 0.80 mm; PLLA/P4HB: 19.50 ± 1.13 mm) were observed in either stent group.

**Table 1:** Luminal patency of the vessel at the distal and proximal ends of the stent (inner vessel diameter, ID_vessel_). Data are presented as mean value±SD. The Kruskal-Wallis test was used to compare groups

		316L	316L + atorvastatin	PLLA/P4HB	PLLA/P4HB + atorvastatin	*p*-Value
ID_vessel_ (mm)	Distal	5.20 ± 0.35	5.42 ± 0.38	5.38 ± 0.63	5.60 ± 0.52	0.638
	Proximal	5.22 ± 0.38	5.22 ± 0.72	4.74 ± 0.45	4.86 ± 0.25	0.302

## Discussion

In this study, biodegradable PLLA/P4HB stents were successfully implanted in the CCA of domestic swine using an endovascular approach. It was possible to show the functionality of the PLLA/P4HB stent by direct comparison with a standard permanent 316L BMS.

The advantages of endovascular management of vascular disease compared with open surgery include reduced mortality and a lower complication rate [3]. The finality of using permanent stents has hitherto represented a problem in interventional treatment with stent placement. Once implanted, they are difficult to remove and, in contrast to biodegradable stents, make surgical intervention in the relevant vessel segment challenging. As lifelong foreign bodies, they continuously cause local immunological irritation in the vessel wall and the associated onset of in-stent restenosis (ISR) [4]. It is known that this causal inflammatory reaction and its effect on proliferation processes can be reduced by removing the foreign body, whereby the use of biodegradable stents represents an advantage.

There has been significantly less investigation into the clinical use of biodegradable stents in the peripheral vascular system compared with the current status of research relating to the coronary vasculature [5]. To date, they have only been used in infrapopliteal interventions in the peripheral vascular system, since smaller stents in this location need to withstand less stress [6]. Femoropopliteal arteries (superficial femoral and popliteal artery), on the other hand, are exposed to considerable mechanical stresses such as compression, bending and torsion. For this reason, there are varying stent fracture rates in vascular segments depending on stent design, with the result that biodegradable stents have only rarely been used in clinical applications in this region [7].

The present study used direct and indirect imaging methods to visualize the implanted stents. Direct methods permitted visualization of the stents themselves, whilst indirect methods demonstrated surrounding changes caused by the stent, without visualizing the stent itself. The permanent metal stents could be visualized, in both dilated and undilated states, at all times under X-ray fluoroscopy, making it possible to assess them directly and at any time. However, it was not possible to directly visualize the biodegradable polymer stents without X-ray markers due to lack of X-ray density during implantation. Only by inflating the balloon catheter with contrast medium was it possible to indirectly observe deployment behaviour. In this regard, biodegradable polymer stents rely on the use of X-ray markers, e.g. gold (Igaki-Tamai stent) [8] or platinum (bioresorbable vascular scaffold stent) [9]. Due to poor differentiability between polymer and vessel wall, it was not possible to measure the outer diameter of the PLLA/P4HB stents or stented vessels during contrast-enhanced CT. However, it was possible to define the inner diameter from the contrast medium column, and thus determine the distal degree of stenosis according to the NASCET definition [10]. The inner diameter of stent-induced stenosis in the distal (and proximal) vessels was comparable in this study, making the two stent groups equal in terms of distal degree of stenosis. At the same time, it should also be borne in mind that stent inner diameter was used to calculate the degree of stenosis, whereby the wall thickness of the PLLA/P4HB stents is proportionally 1.5 times greater compared with the 316L stents (316L: 160 μm vs. PLLA/P4HB: 250 μm).

The present study showed a significantly lower degree of distal stenosis in animals treated with oral atorvastatin compared with untreated animals in the PLLA/P4HB group at contrast-enhanced CT 4 weeks following implantation. Atorvastatin treatment was able to reduce the distal degree of stenosis in the PLLA/P4HB stent group to that of the metal stent group. Given the high prevalence and incidence of cardiovascular disease, statins play an important role in clinical routine. In addition to their main action, low-density lipoprotein (LDL) cholesterol reduction, statins also stabilize plaque [11] and demonstrably reduce restenosis rates [12]. In an animal study, statin-eluting stents were shown to have a significant effect on neointimal hyperplasia in stented coronary vessels in a swine model. An initial clinical study, however, showed statin-eluting stents to cause higher than expected neointimal proliferation [14]. According to the results of this study, biodegradable statin-eluting stents represent a promising opportunity in terms of further experimental investigations to increase drug concentrations at the implantation site and further minimize adverse side effects.

All experimental animals received dual oral anticoagulation with ASA and clopidogrel prior to surgery. The literature reports that this approach resulted in a reduction in occlusion rates in percutaneous transluminal coronary angioplasty (PTCA) with BMS [15], leading to dual antiplatelet therapy becoming established as the standard procedure in coronary interventions with stents [15]. Although studies to assess anticoagulation in stent implantation in the peripheral vasculature are scant, they suggest success similar to that seen in the coronary system [16]. Indeed, our model also showed no occlusion in stented vessels at 4 weeks under this form of dual anticoagulation therapy, as did earlier experiments conducted by our working group [17, 18].

To date, the polymeric stent used here is not sterilized, but rather only disinfected according to the study by Tamai et al. [8]. Various sterilization procedures can have a critical effect on the mechanical properties of the stent. Grabow et al. [19] discovered that sterilization methods using beta and gamma radiation cause a marked reduction in molecular weight and mechanical properties, while increasing crystallinity.

No specially designed balloon catheters to dilate the biodegradable PLLA/P4HB stents were available for the purposes of this study, whereas the 316L stents were available as commercial stent-balloon catheter systems. Therefore, balloon catheters for PLLA/P4HB stent implantation needed to be predilated extracorporeally after the stents had been mounted. Despite the demonstrated feasibility of advancing the predilated balloon catheter over an 8-F sheath with a rotating hemostasis valve for manual rotation (Tuohy-Borst) without removing the mounted stent, the development of a dedicated stent delivery system would make predilatation obsolete and contribute significantly to reducing the sheath diameter required.

## Limitations

All materials used for stent implantation were used in the same way as in humans. This is possible due to the comparable anatomical proportions and physiology of the cardiovascular system in humans and swine [20]. A limitation of the study lies in the fact that stents were implanted in healthy, non-arteriosclerotic vessels; therefore, it is possible that the conclusions drawn here are only valid under the conditions presented.

## Conclusion

- The new biodegradable PLLA/P4HB stent demonstrated that it can be used in endovascular applications and that it is suitable for long-term testing in the established porcine model.
- The future incorporation of biologically active substances aimed at suppressing neointimal hyperplasia could further reduce restenosis rates.
- Overall, the PLLA/P4HB stent represents a promising approach to solving existing problems in the use of permanent stents. Restitutio ad integrum is only achievable if a stent is completely degraded.
